# Facial femininity of potential rivals predicts jealousy in both heterosexual and lesbian women

**DOI:** 10.1038/s41598-025-27643-0

**Published:** 2025-12-17

**Authors:** Junzhi Dong, Benedict C. Jones, Esperanza Miyake, Victor K. M. Shiramizu

**Affiliations:** 1 Department of Psychological Sciences & Health, University of Strathclyde, Glasgow, UK; 2 Journalism, Media and Communication, Department of Humanities, University of Strathclyde, Glasgow, UK

**Keywords:** Psychology, Human behaviour

## Abstract

Individuals displaying cues of higher mate value (e.g., attractive characteristics) are thought to represent a greater threat to pair bonds and, consequently, elicit greater jealousy. Previous studies reporting that more feminine potential rivals elicit greater jealousy in women used stimuli in which feminine characteristics were experimentally manipulated and assessed jealousy using forced-choice methods. However, this method for assessing perceptions has recently been criticised for lacking ecological validity. Consequently, in the current study, women rated how jealous they would feel if rivals depicted in natural (i.e., unmanipulated) face photographs were flirting with their romantic partner. Facial femininity was assessed using objective analyses of shape and via third-party ratings of facial femininity. For heterosexual participants, women reported significantly greater jealousy when imagining more feminine women flirting with their romantic partner. This pattern of results was also seen for lesbian participants, although the correlation between rival femininity and jealousy was significantly weaker for lesbian participants than it was for heterosexual participants. Collectively, these results present further evidence that facial femininity of potential rivals influences women’s reported jealousy, particularly in heterosexual women, and is further evidence for the proposal that putative markers of the mate value of rivals play a role in women’s jealousy.

## Introduction

Many researchers have argued that individuals displaying cues of higher mate value (e.g., attractive physical characteristics) represent a greater threat to pair bonds and, consequently, elicit greater jealousy^1–4^. Consistent with this proposal, women report experiencing greater jealousy when imagining women with more feminine facial characteristics flirting with their romantic partner than when imagining women with less feminine facial characteristics flirting with their romantic partner^5–7^. Similar results have been reported for jealousy and feminine characteristics in women’s voices and body shapes^5,7,8^.

A limitation of the studies described above is that they all employed stimuli in which feminine characteristics were experimentally manipulated and assessed jealousy using forced-choice tasks in which women were presented versions of the same women (e.g., high- and low-femininity versions) and instructed to choose the version that elicited greater jealousy. However, many researchers have recently criticised this method for assessing responses to social stimuli because it has low ecological validity^9–16^. Indeed, several recent studies have demonstrated that effects obtained using this method are typically considerably smaller (and often not significant) when natural (i.e., unmanipulated) stimuli are rated individually^9–12,17–19^. This issue raises questions about the putative link between women’s jealousy and the femininity of potential rivals claimed in previous studies.

In light of the above, we tested for possible interrelationships among (1) women’s reported jealousy when viewing natural (i.e., unmanipulated) female faces, (2) an objective measure of the femininity of those faces’ shapes, and (3) ratings of those faces’ femininity. We included both objective and subjective measures of facial femininity because some previous studies have reported that femininity ratings are more strongly correlated with impressions of female faces than are objective measures (see, e.g. Rhodes^20^ for a meta-analytic review). Dijkstra and Buunk^3^ reported that reading vignettes describing physically attractive rivals elicited greater jealousy in heterosexual than lesbian women. Given positive correlations between physical attractiveness and women’s facial femininity (for a recent review, see Kleisner et al.^21^, Dijkstra and Buunk’s^3^ results predict that correlations between femininity and reported jealousy would be stronger in heterosexual than lesbian women. Consequently, we tested both heterosexual and lesbian women in our study. A weaker effect of rival femininity on lesbian women’s jealousy could occur for many reasons, including (but not limited to) femininity being less strongly linked to lesbian women’s assessments of female attractiveness than it is to heterosexual women’s assessments of female attractiveness^22^, lesbian women’s assessments of feminine rivals being influenced by impressions of feminine rivals suitability as replacements for their current partners as well as potential threats to the stability of the current relationship, and/or lesbian women employing partner-retention strategies that are different to those employed by heterosexual women^23^. Including lesbian women in the current study also presents an opportunity to investigate the extent to which previous findings for jealousy and femininity^5–7^ generalise beyond heterosexual individuals^3^.

## Methods

### Ethics

All aspects of the research were approved by the Department of Psychological Sciences and Health (University of Strathclyde) Ethics Committee (application number 29.13.11.2023). All methods were carried out in accordance with relevant guidelines and regulations all participants provided informed consent.

### Stimuli

Stimuli were face images of 50 white women (mean age = 24.3 years, SD = 4.01 years) from an open-access image database^24^. No female images in this open-access face set were excluded. Images showed women posed front-on to the camera, with direct gaze, and with neutral expressions. Images were standardized on pupil position and clothing was masked prior to rating. The images we used are publicly available at https://osf.io/a3947/.

### Jealousy ratings

The 50 female face images were presented in a fully randomised order to 51 heterosexual women (mean age = 29.53 years, SD = 4.00 years) and 49 lesbian women (mean age = 28.12 years, SD = 4.53 years) in an online jealousy-rating task. These participants were instructed to “Imagine this person was flirting with your romantic partner (if you do not currently have a romantic partner, imagine that you do have one). How jealous would you be? (1 = not very jealous, 7 = very jealous).” These instructions were adapted from those used by Lei et al.^6^ and O’Connor and Feinberg^7^ to assess responses to experimentally manipulated stimuli presented in a forced-choice task. Cronbach’s alphas were high for jealousy ratings made by both groups of participants (both Cronbach’s alphas > 0.93). Jealousy ratings were collected using Qualtrics and participants were recruited via Prolific. Participant sexual orientation was assessed via self-reported orientation identity, all participants were resident in the UK, and all participants reported having English as their first language.

### Femininity ratings

The 50 female face images were presented in a fully randomised order to 30 heterosexual women (mean age = 23.57 years, SD = 5.46 years) and 30 lesbian women (mean age = 23.28 years, SD = 4.46 years) in an online femininity-rating task. Participants were instructed to rate the femininity of each woman shown using a 7-point scale (1 = much less feminine than average, 7 = much more feminine than average).” Cronbach’s alphas were high for femininity ratings made by both groups of participants (both Cronbach’s alphas > 0.92). Because femininity ratings made by heterosexual (M = 3.56, SD = 1.51) and lesbian (M = 3.44, SD = 1.66) participants were strongly positively correlated (*r* =.89, *N* = 50, *p* <.001), we calculated the average femininity rating given to each face from all participants and used those mean femininity ratings in subsequent analyses (M = 3.50, SD = 1.59). Femininity ratings were collected using Experimentum^25^ and participants were recruited by following links on social media. Participant sexual orientation was assessed via self-reported orientation identity.

### Measuring femininity of face shape

Face-shape femininity was objectively assessed for each of the 50 female face images using the facefuns package^26^ in R^27^. This method has been used to assess face-shape femininity in many previous studies^9,17,18,28–31^. Shape components were first derived from Principal Component Analysis (PCA) of 132 Procrustes-aligned landmark points (see Holzleitner et al.^30^, for a diagram showing these facial landmarks) on each of the 50 female faces. Masculinity scores were then calculated for each image using a vector analysis method^9,10,12–15,17,18,28–31^. This method uses the shape principal components to locate each face on a female-male continuum, defined by calculating the average shape information for the 50 white female faces presented in the study and the average shape information for 50 white male faces taken from the same open-access image set^24^. Femininity scores were then derived by projecting each image onto this male-female vector. Higher scores indicate more feminine face shapes. No scores were more than three standard deviations from the mean (i.e., there were no extreme values).

## Results

All analyses were carried out using R^27^ with the kableExtra 1.3.4^32^, lme4^33^ lmerTest 3.1-3.1.1^34^, jtools 2.2.3^35^, psych 2.2.5^36^, and tidyverse 1.3.1^37^ packages. All data, full outputs, and analysis code are publicly available on the Open Science Framework (https://osf.io/v67mk/). Femininity face-shape scores and rated femininity were positively and significantly correlated (*r* =.53, *N* = 50, *p* <.001). Examples of the most and least feminine face images from the sample are shown on the Open Science Framework (https://osf.io/v67mk/).

We analysed jealousy ratings using a linear mixed effects model. In the first model, jealousy ratings were the dependent variable, with shape femininity scores (the facial-metric assessment of shape femininity converted to z scores), participant sexual orientation (effect coded so that heterosexual participants corresponded to −0.5 and lesbian participants corresponded to + 0.5), and the interaction between shape femininity scores and participant sexual orientation as predictors. The model also included by-rater and by-stimulus random intercepts, by-rater random slopes for shape femininity scores, and by-stimulus random slopes for participant sexual orientation. Results of this analysis are summarised in Table 1. There was a significant positive effect of shape femininity scores, indicating that participants reported greater jealousy in response to more feminine women. The interaction between shape femininity scores and participant sexual orientation was also significant. Pseudo R squared (total) for this model was 0.63.

**Table 1:** Summary of results of analysis testing for relationships between shape femininity scores and reported jealousy.

	Estimate	SE	t value	df	*p* value
Intercept	2.398	0.124	19.323	144.578	< 0.001
Participant sexual orientation	−0.363	0.212	−1.714	102.024	0.090
Face-shape femininity	0.232	0.070	3.340	55.053	0.002
Participant sexual orientation x face-shape femininity	−0.102	0.047	−2.149	83.655	0.035

To interpret the significant interaction between shape femininity scores and participant sexual orientation, we repeated the analysis described above, this time analysing jealousy ratings by heterosexual and lesbian participants in separate models. The positive effect of shape femininity scores was significant for both heterosexual participants (estimate = 0.283, SE = 0.081, t = 3.495, df = 61.432, *p* =.001) and lesbian participants (estimate = 0.181, SE = 0.065, t = 2.787, df = 54.048, *p* =.007), but was significantly stronger for heterosexual participants.

Next, we repeated our initial analysis, this time replacing shape femininity scores with rated femininity. Rated femininity was z-scored prior to analysis. Results of this analysis are summarised in Table 2. There was a significant positive effect of rated femininity, indicating that participants reported greater jealousy in response to more feminine women. The interaction between rated femininity and participant sexual orientation was also significant. Pseudo R squared (total) for this model was 0.64.

**Table 2:** Summary of results of analysis testing for relationships between rated femininity and reported jealousy.

	Estimate	SE	t value	df	*p* value
Intercept	2.398	0.116	20.665	133.238	< 0.001
Participant sexual orientation	−0.363	0.211	−1.721	100.632	0.088
Rated femininity	0.389	0.057	6.874	71.485	< 0.001
Participant sexual orientation x rated femininity	−0.169	0.057	−2.979	95.769	0.004

To interpret the significant interaction between rated femininity and participant sexual orientation, we repeated this analysis, this time analysing jealousy ratings by heterosexual and lesbian participants in separate models. The positive effect of rated femininity was significant for both heterosexual participants (estimate = 0.474, SE = 0.067, t = 7.115, df = 82.064, *p* <.001) and lesbian participants (estimate = 0.305, SE = 0.060, t = 5.090, df = 69.409, *p* <.001), but stronger for heterosexual participants.

Sensitivity power simulations showed that both analyses had 80% power to detect small-sized effects for the interaction between femininity and participant sexual orientation. Full results and details of these simulations are publicly available at https://osf.io/v67mk/.

## Discussion

The current study investigated how measured femininity of rivals’ face shapes and rated femininity of rivals’ faces were related to reported jealousy when heterosexual and lesbian women imagined the potential rivals flirting with their romantic partner. Our analyses showed that heterosexual women reported significantly greater jealousy when imagining women with more feminine face shapes flirting with their romantic partners. This same pattern of results was also observed when femininity of potential rivals was assessed via third-party ratings of facial femininity. Both results are consistent with previous research in which heterosexual women reported greater jealousy of more feminine rivals when feminine characteristics were experimentally manipulated in face images^5–7^, body images^5^, or voice recordings^7,8^. Our results are also consistent with the proposal that individuals displaying cues of higher mate value (e.g., attractive physical characteristics) represent a greater threat to pair bonds and, consequently, elicit greater jealousy^1–4^. Importantly, our study is the first to show that previously reported results for manipulated facial femininity and jealousy extend to jealousy ratings of natural (i.e., unmanipulated) faces images that vary on multiple dimensions simultaneously.

The pattern of results that we observed for lesbian women’s jealousy ratings was very similar to the pattern that we observed for heterosexual women (i.e., more feminine potential rivals elicited greater jealousy). However, for lesbian women, the correlation between reported jealousy and femininity was significantly weaker than the corresponding correlation that we observed for heterosexual women. This weaker effect of facial femininity on lesbian women’s reported jealousy is consistent with Dijkstra and Buunk^3^, who found that vignettes reporting physically attractive women elicited greater jealousy in heterosexual women than in lesbian women. It is also consistent with Shiramizu et al.^22^, who reported that lesbian women showed significantly weaker preferences for female faces with feminine characteristics than did heterosexual women. The weaker correlation between femininity of potential rivals and jealousy reported by lesbian participants than reported by heterosexual women may reflect greater variability in femininity preferences of lesbian women’s partners (women attracted to women) than heterosexual women’s partners (men attracted to women, see, e.g., Bailey et al.^38^.

As mentioned in our Introduction, previous studies testing for possible effects of facial femininity on women’s reported jealousy used stimuli in which feminine characteristics were experimentally manipulated and assessed jealousy using forced-choice tasks^5–7^. This method for assessing responses to facial cues has been criticised for lacking ecological validity^11,16^ and many recent studies have reported that results obtained using this method are qualitatively different to those obtained when natural (i.e., unmanipulated) face images are rated^9,19^. By contrast with this pattern of results, however, the current study observed effects of facial femininity on reported jealousy that were similar to those obtained using the forced-choice method in previous studies^5–7^. Thus, our findings raise the possibility that the differences between results obtained using natural (i.e., unmanipulated) and experimentally manipulated face stimuli may be more nuanced than some previous research has suggested. At least for jealousy and facial femininity, these two different methods appear to show similar patterns of results.

The current study compared the effect of rival femininity on jealousy perceptions in heterosexual and lesbian women. Bisexual and pansexual women were not included in the current study. Future research employing more continuous measures of sexual orientation that considered a wider range of orientations may be a useful avenue for future research. Indeed, such work may help clarify precisely why sexual orientation appears to moderate the effect of femininity on jealousy perceptions, as discussed in our Introduction. Testing whether other factors, such as participant relationship status, also moderate the observed effects of femininity on jealousy perceptions might also be a potentially useful direction for future research.

Our findings for rival femininity and reported jealousy were observed in a sample of English-speaking residents in the UK. Consequently, it remains unclear whether our findings would necessarily generalise to other types of samples (e.g., samples recruited from different countries and cultures). Similarly, the stimuli in our study were all white faces, meaning that it is also unclear whether our findings would generalise to stimuli of other ethnicities or image sets with diverse ethnicity. Addressing these constraints on the generalisability of our findings is an important topic for future research.

Previous research linking facial femininity to women’s jealousy used manipulated stimuli and forced-choice methods^5–7^ that have recently been criticised for having poor ecological validity^11,16^. Here we found that jealousy ratings of natural (i.e., unmanipulated) face images were positively correlated with femininity of face shape and that this correlation was stronger for heterosexual women than lesbian participants. These findings underline the importance of rivals’ facial femininity for women’s jealousy and suggest that previous findings were not simply an artefact of the methodologies employed.

## Data Availability

All data, full outputs, and analysis code are publicly available on the Open Science Framework (https://osf.io/v67mk/).
